# The student opportunities for AIDS/HIV research program: promoting public health leadership and transformation for undergraduate students through a principles-driven, cohort-based model

**DOI:** 10.3389/fpubh.2025.1601175

**Published:** 2025-07-30

**Authors:** Gabriel Lee Johnson, Leah C. Neubauer, Heidi Bennett, Andrea Bolivar, Anna R. Kirkland, Gary W. Harper

**Affiliations:** ^1^School of Social Work, Michigan State University, East Lansing, MI, United States; ^2^School of Public Health, University of Michigan, Ann Arbor, MI, United States; ^3^School of Education, University of Michigan, Ann Arbor, MI, United States; ^4^Department of Women's and Gender Studies, College of Literature, Science and the Arts, University of Michigan, Ann Arbor, MI, United States

**Keywords:** mentoring, HIV, leadership, feminist research, public health, undergraduate research education

## Abstract

**Introduction:**

Undergraduate public health degrees have grown over 1,100% over the past 20 years, not including interdisciplinary scholars who are interested and do not major in the field, marking an opportunity for proactive public health leadership in this burgeoning group of people with potential futures as public health leaders. The Students Opportunities for AIDS/HIV Research Program (SOAR) is a 2-year program funded by the National Institute of Mental Health (NIMH), providing research training and leadership development to historically underrepresented college undergraduates as formerly defined by NIMH. SOAR prepares students for a future as interdisciplinary HIV researchers and leaders in diverse disciplines, including public health. Rooted in critical feminist values and utilizing a cohort model, a high impact practice (HIP; Opacich), SOAR demonstrates tremendous potential for developing collaborative, transformational leaders (Teitel) and an example of a multi-tiered mentorship model.

**Methods:**

SOAR is housed in the Institute for Research on Women and Gender, an interdisciplinary unit of the University’s Office of the Vice President for Research and is operated in partnership with a CEPH-accredited School of Public Health at an elite predominately white institution (PWI). The program is grounded in guiding principles that center the foundational work of Black feminist scholars and activists, as well as the scholarship of Transformative Education (hooks). The development of the various components of the program were guided by the expanded Social Cognitive Career Theory (eSCCT) pedagogical frameworks of cohort models (Opacich) and mentorship ecosystems (Endo).

**Results:**

To date, three out of the four cohorts have graduated from the SOAR program, with 90% matriculating into advanced degree programs. SOAR scholars have also co-authored 32 peer reviewed articles and delivered over 80 presentations or panels at conferences. SOAR scholars have matriculated into a diverse array of disciplinary programs, moving towards their next step towards becoming HIV and public health researchers and leaders.

**Discussion:**

Approaches to developing cohorts were employed in addition to leveraging key critical, feminist approaches including embracing difference as key to cohort and leadership development, identifying key collective struggles to build cohesion, fostering a community of care, and embracing diversity across numerous social and developmental locations within the cohort. The SOAR program provides not only key ley lessons outlined, but also an example for future programs to follow and engage in early HIV and public health research leadership development at the undergraduate level.

## Introduction

Undergraduate public health degree programs have grown over 1,000% in the past 20 years (1). These programs educate students who major in public health as well as those in other disciplines who participate in public health courses and extracurricular learning activities. Public health is a flexible and dynamic degree, which welcomes a diversity of disciplines and training into the field and a great opportunity for undergraduates to enter this field. This expansion of public health education and training to undergraduate students throughout a range of academic majors aligns with the 2003 Institute of Medicine (IOM) Committee on Educating Public Health Professionals for the 21st Century report, which called for not only a well-educated public health workforce but also an educated citizenry (2).

However, many who pursue undergraduate public health degrees do not attend graduate school (1). The expansion of undergraduate public health education marks an opportunity for proactive public health leadership development in this burgeoning group of students with potential futures as public health leaders (3). Existing literature on public health leadership has focused primarily on graduate training (4–6) which presents missed opportunities for earlier leadership development as part of undergraduate public health training. Elaine Auld et al. (7), citing gender disparities in leadership, recommends the integration of leadership skills in the undergraduate curriculum including examples of female health education trailblazers who advocated for social and racial justice.

This paper describes an undergraduate public health research education pipeline program focused on supporting undergraduate students in completing their bachelor’s degree (both those who do and do not major in public health) and then gaining acceptance into a graduate program. Further, it details critical practices which support and develop the next generation of public health leaders through a research pipeline program to graduate study. Anchoring public health research development during undergraduate education is a proactive step to grow public health leadership and develop a more sustainable and dynamic foundation for their career development. Our work with undergraduate students expands notions of *who may be a public health leader* and engages potential leaders early at the undergraduate level. The Student Opportunities for AIDS/HIV Research (SOAR) program prepares students for public health leadership early, during undergraduate education as compared to during graduate school or in the workforce, diversifying research perspectives in public health through interdisciplinary training.

SOAR is a two-year intensive academic, research mentoring, and leadership development experience for undergraduate students in their junior and senior years grounded in feminist intersectional theory and praxis. The program was formerly funded by the National Institute of Mental Health and the Office of Behavioral and Social Science Research at the National Institutes of Health (NIH; #R25MH126703) and has continued despite early termination of its federal funding. It provides foundational training and leadership development to undergraduate students who are interested in a career in behavioral social science research (BSSR) related to HIV, preparing them for a future as interdisciplinary public health leaders and researchers. SOAR utilizes a holistic education approach (8) to learning that incorporates an array of active and participatory teaching and learning strategies that are focused on developing the “whole person” as opposed to a traditional linear approach to academic achievement.

## The foundations of SOAR

SOAR is housed in the Institute for Research on Women and Gender, an interdisciplinary unit of the University’s Office of the Vice President for Research and is operated in partnership with a CEPH-accredited School of Public Health at an elite predominately white institution (PWI). The program is grounded in guiding principles that center the foundational work of Black feminist scholars and activists, as well as the scholarship of Transformative Education (9–17). The development of the various components of the program were guided by the expanded Social Cognitive Career Theory (eSCCT) (18–21), pedagogical frameworks of cohort models (22) and mentorship ecosystems (23).

### Selecting SOAR students

SOAR is a two-year program that aims to (a) support students in the completion of their undergraduate degree and (b) prepare students for doctoral-level graduate education and eventual research careers in HIV-related BSSR, with a preferred focus on HIV prevention and treatment within sexual and gender minority communities. The program was developed to focus on students from historically marginalized and underrepresented groups (URG) as formerly defined by the NIH. At the time of our proposal and funding, NIH defined underrepresented minority students as those who identify as (a) Black/African American, Hispanic/Latino, American Indian/Alaska Native, Native Hawaiian or other Pacific Islander; or (b) an individual with a disability; or (c) an individual from a disadvantaged background (at least two of the following: were or currently homeless, were or currently in foster care, were eligible for federal free or reduced lunch for two or more years, had no parents or legal guardians who have completed a bachelor’s degree, were or currently eligible for Pell grants, or received support from the Special Supplemental Nutrition program for women, infants, and children as a parent or child). Given that most people at risk for or living with HIV in the US identify as sexual and/or gender minority (SGM) people, SOAR focuses on providing mentored research experiences with faculty members who conduct HIV-focused BSSR with SGM communities. Given this, SOAR also attracted a substantial number of undergraduate students who also identify as SGM.

SOAR includes a multi-step admissions process. To participate in SOAR, students must apply for acceptance before entering their junior year and have at least 2 years of school remaining. Students must demonstrate an interest in BSSR related to HIV or populations disproportionately impacted by HIV. Promotion of SOAR has included advertising the program to a diverse array of majors via email listservs and social media, tabling at student events, speaking in general education courses, and advertising in the campus newspaper. Although we did conduct focused outreach to student groups with membership from under-represented student communities, SOAR is open to all undergraduate students at the University.

The core of the application is a series of short essay questions in which students describe their demonstration of leadership potential, relevance of HIV BSSR to their future goals, and why SOAR would be a good fit for them. The SOAR Co-Directors then reviewed all applicants and selected the most qualified applicants for an interview with two members of the SOAR leadership team to ensure that there was a diversity of perspectives for each applicant. After interviews were conducted, members of the leadership team met to make decisions on which applicants were either admitted, waitlisted, or rejected. The decision to admit applicants was made holistically, with the goal of creating an interdisciplinary and cohesive cohort rather than singular characteristics guiding admission (i.e., high grade point average, specific academic major, research experience). After finalizing decisions, emails were sent to admitted students requesting decision on their admission to SOAR. If applicants declined, then members on the waitlist were offered program admission.

### The SOAR program guiding principles

SOAR, program administrative leadership established guiding principles to shape and clarify key beliefs and values. The principles emerged from a group reflection on SOAR aims and activities following the initial year of the program. Although they were formally articulated after the program was already in operation, the group felt they had been implicit pillars of the program since its inception thus we present them first since they serve as the grounding foundation for all that we do in the SOAR program. The seven guiding principles for SOAR were informed by the scholarship of Transformative Education and Black feminist scholars and activists (9–17) and adapted from D’Ignazio & Klein (24) principles for equitable and actionable COVID-19 data. The principles below serve as a guiding philosophy for program actions and decisions. These principles were shared with SOAR scholars, faculty and doctoral mentors to clarify the norms, beliefs, values, and expectations of the SOAR program.

1. Examine Systems of Power: We strive to examine systems of structural privilege and structural oppression and investigate how they manifest at different levels. This involves naming and understanding the realities of systems of racism, sexism, classism, ableism, colonialism, heterosexism, and cissexism and how they compound to negatively influence the lives of oppressed communities.
2. Value Multiple Forms of Knowledge: We value subjugated knowledge and various ways of knowing, and challenge dominant notions of objectivity through our program activities. This is reflected in how we conduct research and prioritize the lived experiences of students. In practice, we privilege communal knowledge, especially that which exists within the communities with which we work.
3. Challenge Unequal Power Structures: We recognize, analyze and challenge power dynamics that exist within multiple systems and structures of influence, both visible and invisible. This involves discussing the visibility of power and maintaining vigilance to redress power imbalances.
4. Support People Holistically: We support our SOAR community members as living, feeling bodies in the world. We elevate emotion and embodiment through attention to space, food, social activities, levels of physicality, and differing values. We celebrate and value familial and family-of-choice dynamics and responsibilities.
5. Rethink Binaries: We promote thinking outside of binaries by allowing space for individuals to exist as whole persons who occupy liminal spaces. This involves creating space for other voices to be heard and to consider different multiple ways of thinking about issues that arise in HIV research and practice, as well as in other related areas.
6. Celebrate Diverse Journeys: We celebrate the multiple paths, backgrounds and life histories of our SOAR community members. We foster respect and open-mindedness regarding diverse life journeys and celebrate both our differences and our common interests.
7. Make Labor Visible: We strive to identify, acknowledge, and value all forms of labor, including emotional labor and other forms of supportive labor that are often under-compensated. In managing the resources of the SOAR program justly, we focus on adequately compensating people for their work and avoiding overwork and burnout.

### SOAR theoretical framework

**Figure 1:**
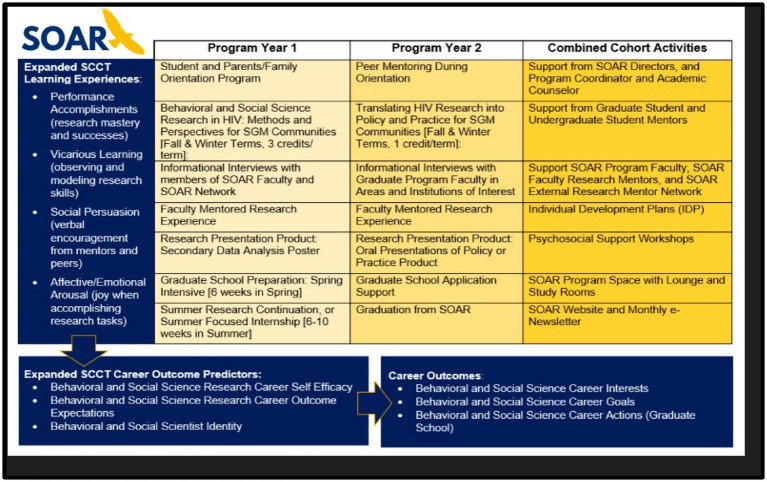
Diagram of SOAR components and linked eSCCT outcomes.

Figure 1 details the original key SOAR activities proposed in which students engaged throughout the duration of the program, including how certain activities align with the expanded Social Cognitive Career Theory (eSCCT) (18–21) which informed the inclusion of program structures and activities. Through the various components of the program, SOAR students were engaged in BSSR career performance activities and accomplishments. For example, they worked with a research team conducting various research-related activities as part of their Mentored Research experience and conducted secondary analyses of BSSR data and presented their findings at our annual Dr. John Lamont Peterson SOAR Research Symposium. Students learned vicariously by observing graduate and professional researchers in their mentored research experience. Scholars also felt encouraged receiving praise from faculty and high school peers. Lastly, affective/emotional arousal regarding BSSR careers (e.g., feelings of excitement when mastering new material or research skills, and pride when presenting research findings to others).

### SOAR pedagogical frameworks

The program was delivered using two pedagogical frameworks that support the focus on holistic learning within educational and professional development. The first is a *cohort model*, a high-impact practice (22), which holds tremendous potential for developing collaborative, transformational leaders (25, 26). The second is *a mentorship ecosystem, which emphasizes a dynamic, values-centered system approach* (23), that decenters a singular mentor but cultivates mentors at the faculty and doctoral level to support students’ professional and academic goals. In implementing these pedagogical frameworks, multiple components were employed to support matriculation of SOAR scholars into graduate programs and support development of scholars as HIV-focused behavioral social science researchers.

#### Cohorts and courses

SOAR scholars were required to take two yearlong courses together, one in the first year and another the second year. The first-year course focused on highlighting feminist perspectives and methods in HIV BSSR. Additionally, the first-year course provided SOAR scholars support with composing their research posters in preparation for the SOAR Symposium. The course was 2 days a week with a tenure-track professor in the Women and Gender Studies Department and whose background is in cultural anthropology. The second-year course was focused on translating HIV research to policy and practice, meeting 1 day a week and was taught by a full professor in the School of Public Health whose background is in the wellbeing of sexual and gender minorities and HIV prevention. The second-year course later changed structure, with the first semester dedicated to refining graduate school materials and the second semester dedicated to translating HIV research to policy, which was applied in a brief academic presentation students gave during the SOAR symposium, which is described later in this section. Below we provide brief descriptions of SOAR program components, which primarily occurred within the first year of the program except for graduate school preparation, application, and translating HIV research to policy, which occurred within the second-year course described earlier.

The cohort model is considered a high-impact practice because of its positive effects on student engagement, learning, and success. It promotes collaboration, builds strong relationships, and fosters a sense of community, all of which are linked to improved academic outcomes. In addition, Opacich (22) talks about how a cohort model in public health training at the undergraduate level can help to create a community of learners. As it pertained to SOAR, the cohort model was implemented through the required courses, and numerous components that focuses on creating this community of learners. Later in this paper discusses how specific components facilitated students understanding of each other’s strengths, needs, and support each other.

#### Mentor ecosystem

The SOAR mentorship ecosystem was designed to promote multiple mentors at varying stages of becoming social behavioral scientist. Faculty were responsible for providing mentored research experience, SOAR scholars were also paired with doctoral students, who provided insight and guidance on the journey to a doctoral degree from a lived experience perspective as a near-peer mentor. Peer mentoring occurred organically and through curated activities designed to build camaraderie. Peer connection across cohorts also normalized the feelings of students in the first year of the program, creating a sense of community and shared experience.

SOAR scholars were provided with two formal mentors: the mentored research experience (MRE) with a faculty member and less structured doctoral mentor. The process for matching faculty MRE mentors was quite extensive to ensure a beneficial match between mentors and mentees. After students accepted a position as a SOAR Scholar near the end of their sophomore year, they were provided with standardized overviews of all the potential mentors for their MRE, and faculty were provided access to the applications submitted by all accepted SOAR students. Both students and faculty members then selected three people with whom they wanted to complete their MRE and ranked them in their order of preference on an online ranking form. If a student or faculty mentor had no preferences, they stated so on the form. An administrative team member then scheduled all the interviews, after which time both students and faculty mentors submitted a revised ranked list of their preferred MRE partner. Based on those final requests, the SOAR leadership team matched SOAR students with their faculty mentor.

SOAR scholars are paired with doctoral student mentors by the end of the first semester of the program. The matching process was far less extensive than that for the faculty members and was based heavily on finding a doctoral mentor who possessed characteristics that the SOAR Scholar wished to have in this mentor. For some the match was based on disciplinary fields and areas of study, while for others it was based on shared social categories or lived experiences. The relationship between doctoral mentors and SOAR scholars was less prescriptive, as relationships included problem solving challenges related to scholars’ overall college experience and assistance with graduate school applications. Both faculty and doctoral mentors were provided training and mentoring best practices adapted from the “Entering Mentoring” curriculum from the Center for the Improvement of Mentored Experiences in Research (27). Faculty mentors were given academic year effort on the grant and doctoral mentors were financially compensated.

## Other critical components of SOAR

### Mentored research experience

In addition to the first-year course which utilizes the cohort model to build a well-connected and supportive learning community among students and provides them with critical foundational knowledge and skills related to BSSR and HIV, another critical component of the SOAR program in the first year of the program is the Mentored Research Experience (MRE). Students participate in 10 h of MRE-related activities (e.g., readings, meetings, trainings, etc.), and receive payment for their time. They join research team meetings and meet individually with their MRE mentor for 30–60 min each week. The MRE faculty mentors also meet twice a semester with one of the SOAR Co-Directors to discuss student progress, and to discuss any potential challenges that may arise.

The MRE is not a traditional research assistantship where students complete hours conducting specific research tasks such as entering or coding data, but rather a research apprenticeship where the students learn how to become a BSSR focused on HIV. The undergraduate student becomes an active member of the mentor’s research team, and the mentor provides the student with readings, activities, and online learning related to various aspects of the research process such as research design, research ethics and Institutional Review Board (IRB) policies, research questions/hypotheses, methods, and dissemination. Faculty mentors are provided with a SOAR Program Comprehensive Mentor Syllabus which provides month-by-month integrated guidance for all aspects of the mentees learning activities, including specific information about recommended content and activities being conducted at the SOAR program level, in the SOAR academic course, and in the MRE. The faculty mentor also discusses paths to graduate school, career objectives, and introduces their mentee to other HIV-related BSSR.

Throughout the year-long MRE, the SOAR scholar and mentor develop a research question or research hypotheses that can be examined using secondary data. They work together throughout the semester to cultivate a BSSR HIV-related analysis project that is doable during the year, and that is socially relevant. The project is conducted by the student with assistance from the mentor and other research team members, and results in an academic poster presentation at the annual SOAR conference described below. Some of the research projects titles that SOAR scholars presented were “Perceived sexual risk of HIV among South Asian gay and bisexual men in the United States,” “Cuts and Community: The impact of barbers and normative beliefs on young Black men’s condom use attitudes,” and “Negative influences of psychosocial factors on HIV prevention and care among gay and bisexual men in Kenya.”

### SOAR symposium

At the end of each year in SOAR, the Dr. John Lamont Peterson SOAR Symposium is held, named after the late HIV scholar and activist who was instrumental in employing critical methods in order to understand key factors to reduce HIV risk, including giving attention to social determinants of HIV infection among young Black gay and bisexual men to reduce racial inequities (28). Symposiums include a keynote presentation from prominent BSSR scholars who address current topics and issues in HIV BSSR and provide insights and stories from their career journeys. They offer advice, support, and encouragement, and remain at the conference all day to allow for more intimate discussions. Central to the SOAR symposium are the research poster session completed by first year SOAR scholars, where they display and discuss their research findings and implications, serving as a culmination of their MRE. In addition, the graduating SOAR scholars present a 7-min HIV-related research or policy oral presentation, or “lightning talk,” based on either policy work they have conducted in their second year in the program or on research data from a continuation of their MRE into the second year. Lightning talks conducted by second year SOAR scholars included topics that advocate for increased training on gender inclusive parent–child sexual health discussions, tailored HIV care during war in Ethiopia, and increasing eligibility and access of pre-exposure prophylaxis to formerly incarcerated heterosexual Black men. The SOAR symposium served both as a culmination of the research and policy-based experiences of each respective year, and a way for both cohorts to close out the year and end of their time in the program.

### SOAR supper

After receiving feedback from the inaugural cohort that more time together to discuss professional development, researcher identity, and career trajectories in a relaxed and open environment, the leadership team developed a series of events called SOAR Suppers. Originally conceived as psychosocial support workshops as indicated in Figure 1, these were monthly dinners hosted by SOAR as loosely facilitated discussions with a member of the SOAR leadership team. Topics included reflecting on the decision to pursue graduate school, navigating imposter syndrome, and other identity development topics. They also serve as another opportunity to build camaraderie and cohesion within the cohort. While SOAR Supper topics were predetermined, they were open to change based on the needs of the group. For example, a session focused on developing graduate school criteria and a preliminary list of schools pivoted to a discussion about internships and aligning your summer experience with professional and academic goals rather than what one’s peers were doing.

### May intensive

Following the end of the first academic year of SOAR, scholars then participated in the May Intensive (formerly called grad school prep), a month-long mini course that focused on the development of graduate school application materials. Students were compensated for their time during the May Intensive and paired with one another and a faculty member to write and revise their graduate school materials, with a focus on the personal statement. May intensive consisted of workshops, as well as guest speakers who were current doctoral students, as well as junior and senior faculty members. Topics ranged from imposter syndrome, perspectives of current and prior admission committee members, and personal and professional biographies. Although the primary focus of the May intensive was to develop a full draft of the personal statement, scholars also drafted a curriculum vitae, shortened list of graduate schools to apply to, statement of purpose, and a list of people from whom they wish to receive a letter of recommendation. Students also had the option to take a GRE preparation course provided by a professional company, and SOAR paid all fees. Following the conclusion of the May intensive, scholars then began their summer experience.

### Summer experience (previously summer research continuation or focused internship)

Prior to the end of the first year of SOAR, scholars participated in a two-month summer BSSR research and/or practice-based experience called the summer experience. In the early days of the program, we encouraged students to apply for external summer internships to build their network of colleagues and mentors working in the HIV and BSSR space. Scholars were given latitude over what they chose as their summer experience if their justification highlighted how the proposed experience aligned with future academic and professional goals. Scholars chose a range of options including continued work with their MRE faculty members to develop manuscripts for publication, external formal research training programs, and policy programs through state governments, to name a few. If summer experiences were not funded through the program or faculty, SOAR then provided funding for scholars for the duration of the summer experience. Previously named the summer focused internship and research continuation, we noticed students felt increased pressure to find and secure prestigious internships rather than secure opportunities aligned with their future goals and desired skills, which prompted us to change this component to the less specific Summer Experience. Additionally, after the first two cohorts of students, we noticed that those students who remained on campus and focused on research activities with their MRE faculty member or other SOAR mentors were able to be more productive at disseminating their research findings. Thus, we moved to a model of having students conduct an 8-week summer research internship at the University with a faculty member of their choosing and are paid for 20 h of research activities a week. This experience is not like the highly structured and holistic training in the MRE but is more like a traditional research assistantship. Following the conclusion of the summer experience, SOAR students, faculty, and staff took a one-month break to rest and recuperate.

## Results

### General outcomes related to transition to public health focused graduate programs

To date, SOAR has enrolled four cohorts, and 100% of students have been accepted into graduate degree programs. See Table 1 for detailed information related to graduate school matriculation of the three graduated cohorts to date. Approximately 62% of the 29 graduated scholars across three cohorts are attending master’s in public health programs. Among all SOAR graduates, 76% have successfully matriculated into public health-related graduate programs with a focus on BSSR (e.g., psychology, social work, and public health) at both the master’s and doctoral level, the next step in their path to public health leadership. Four SOAR scholars (14%) have entered doctoral programs, with two pursuing a PhD in social psychology, one in Asian studies with a focus on HIV globally, and one a law degree. Additionally, one student is currently on an esteemed global fellowship engaging in community-driven health work, and was able to defer their acceptance into an MSW program. SOAR scholars have also contributed to scholarly conversations in HIV research. To date, SOAR scholars who have graduated and are currently in the program have co-authored 31 peer-reviewed publications and given 84 presentations at conferences. SOAR’s distinct approach to developing a HIV BSSR pipeline was key to the program’s success in matriculating 90% of its students into advanced degree programs, with the remaining 10% taking a gap year or fellowship with intentions to pursue an advanced degree.

**Table 1:** Post undergraduate matriculation data of graduated SOAR scholars.

Graduate school degrees	Post undergraduate	Completed graduate degree*
Master’s Degree Program in Public Health (MPH)	62% (18)	33% (6)
Master’s Degree Program in Social Work (MSW)	7% (2)	50% (1)
Master’s Degree Program in Biomedical Engineering (MS)	3% (1)	
Master’s Degree Program in Sociology (MSc)	3% (1)	
Doctoral Degree Program in Social Psychology (PhD)	7% (2)	
Doctoral Degree Program in Humanities with an HIV focus (PhD)	3% (1)	
Juris Doctor Degree Program (JD)	3% (1)	
Global Health Fellowship (deferred admission into master’s degree program)	3% (1)	
Public Health Research/Practice Gap Year (deferred admission into master’s programs)	7% (2)	
Total	100% (29)	24% (7)

*Indicates percentage graduated from program out of total across cohorts who have matriculated into graduate program discipline, all of whom are from the first cohort. Other scholars are currently in graduate program.

## Conclusion

### Key approaches to support public health leadership

SOAR’s focus on HIV and BSSR, rather than a specific discipline area, provided an opportunity to incorporate pillars of public health research, theory and practice, along with a diversity of disciplines at the University to inform a SOAR scholar’s view of public health. What follows are key reflections and examples from programmatic components of SOAR that have supported SOAR’s success in graduate school matriculation with diverse cohorts, facilitating experiences that increased scientist identity and leadership skills.

### Embracing difference through social and developmental diversity

A focus on varying dimensions of diversity (e.g., race, socioeconomic status, disability status, sexual orientation, gender identity, etc.) is important in developing public health leadership and BSSR scholars who can speak to the vast and varied experiences of those disproportionately impacted by HIV and other health inequities. When annually conducting admissions and constructing cohorts, SOAR leadership gave attention to the range of research experiences students had previously, ranging from those who had no research experience at all to research experience every semester in college leading up to applying for SOAR. Consideration was given to how students articulated a professional or academic path for themselves, ranging from students who were very explicit to those who were in the exploratory stage or had not considered a future beyond a bachelor’s degree. Overall, there was a focus on admitting students who aligned with SOAR’s expressed interest in HIV-related BSSR as compared to applicants who had extensive research experience but lacked clear interest in the focus of the SOAR program.

Differences across various dimensions of experience among SOAR cohorts provided opportunity for leadership, skill building, and building confidence in scholars’ identity as a BSSR scientist. The development of cohorts that were diverse in experience and backgrounds provided leadership opportunities among peers. Embracing differences across cohorts, SOAR scholars were able to leverage both their lived and learned experiences. For example, experience with varying types of oppression and hardship differ across individuals and social categories, and through facilitation and centering of lived experiences of SOAR scholars in the first-year course, the instructor brought attention to how skills developed outside of the classroom are necessary to conduct research with key populations disproportionately impacted by HIV. Skills included a critical eye and understanding the core issue of a health inequity, communicating across populations of practitioners and impacted communities, and incorporation of distinct approaches to community involvement in program development and implementation. Some SOAR scholar’s skills were reflected in poster presentations and lightning talks during the SOAR symposium. Beyond distinct experiences held by each SOAR scholar, components such as the SOAR courses, suppers, and the May intensive provided an opportunity to increase cohesion among the cohort and emphasize the importance of leadership as a collective trait and not simply an individual one.

### Collective struggle toward cohesion

The national context described previously that SOAR scholars navigated prior to and during SOAR contributed to increased stress, including discriminatory experiences which were more likely reported among generation Z (born after 1997), sexual and gender minority students, and racially minoritized students (29), all of which comprise the entirety of SOAR scholars. Although SOAR scholars navigated stressful and oppressive context in addition to the stressors of preparing for graduate school and futures in HIV-related BSSR and public health leadership, experiencing these stressors in a small cohort provided opportunities for cohesion among SOAR scholars and the value of collective leadership, or not relying one person to lead.

The stress that occurs while preparing for a future in BSSR-focused HIV research is common; however, its presence in SOAR cohorts along with learning of feminist praxis galvanized into not only increased cohesion among cohorts but also advocacy and action. As students shift from taking the introductory course in their first semester, to preparing their research posters for the symposium, finding summer experiences to provide relevant experience for their next professional steps, and capping off the first year of SOAR with a month-long intensive dedicated to developing graduate school materials, there is a building stress that SOAR scholars experience in the first year. This stress, which numerous SOAR scholars have acknowledged, became an opportunity to strengthen the bonds of the cohorts.

Rather than building smaller factions and not sharing pertinent information that may be helpful to others in their cohort, SOAR scholars instead strengthened and built bonds with one another focused on navigating barriers to their goal of becoming BSSR scholars. Increased cohesion among SOAR scholars often showed up as increased collective action and interdependence, relying less frequently on SOAR leadership and increasingly on one another. At times, the cohorts would advocate for the needs of a few, leveraging the power of the larger collective. For example, instead of allowing one person to advocate for increased economic support for themselves, SOAR scholars collectively advocated to SOAR leadership while holding SOAR leadership accountable to the principles of the program. These bonds were evident and effective in the second year of the program as well, as students shared information about various graduate programs, reviewed each other’s application materials, collaborated on publications, celebrated each other’s graduate school acceptances, and provided much needed support and encouragement as they collectively navigated the demands of completing their undergraduate degree and gaining admission into graduate school.

The development of the collective is incorporated into feminism, focusing on interdependence, rather than independence or isolation, which is often reinforced in highly selective research institutions, a sentiment echoed by SOAR scholars. Group-based components of the program (i.e., first- and second-year course, SOAR Suppers, May Intensive, and Symposium) provided iterative opportunities for increased cohesion among the SOAR cohorts and simultaneously provided opportunity to develop and practice how team-based leadership can be effective in a safe, development-oriented environment.

### Community of care

In addition to group-based components of SOAR that contributed to leadership development and strengthening confidence and skills in conducting research, SOAR provided a community of care that allowed people to feel cared for amid a large public elite university and learn from mistakes throughout their 2 years in the program. Key to building a community of care, or a community where everyone can contribute to the needs of the collective (or in this instance, cohort) and individual well-being, was cultivating a space for students to convene (i.e., SOAR lounge). We designed SOAR not as an organization focused solely on graduate school and research, but as an organic, flexible, and caring organization.

Demonstrating this value occurred throughout both years of the program. In the first-year course the professor frequently supported students’ needs beyond the scope of the course (i.e., self-esteem, family challenges, etc.). This aim also included demonstrating care by establishing and communicating boundaries around how the first-year professor would provide support for SOAR scholars, such as limiting requests for reference letters and setting expectations around communication and response times. Particularly in a program where many shared identities exist between leadership and scholars, implementing boundaries within the context of caring for oneself provides clear examples for the scholars who are embarking on a journey where they may experience burnout. The professor for the second-year course used a flexible active learning format for the class which allowed for individual and group work time and allowed for students to engage in private conversations with the professor in a secure space outside of the classroom when needed. Since the class took place after 5 pm, the professor provided students with either snacks or a full meal and provided celebratory cupcakes or cookies for birthdays and other notable dates or accomplishments, as well as motivational pens and materials. Each class session began with graduate school application updates, life achievement updates, and responses to two questions: (a) what do you need? (b) how can we (students and professor) best support you?

While developing research skills was a primary goal of SOAR, embracing difference, a challenge or struggle most of the cohort can relate to, and developing a community of care were key to developing leadership skills. By developing a cohort that had differences across various backgrounds and skills, the program provided an opportunity for peers to flex distinct leadership capabilities. This development often happened while SOAR scholars were wrestling with the stressful task of preparing for graduate school and within the co-created community of care that was essential to establish early on during their first-year course. Our findings highlight what practices and lessons may be beneficial in developing public health leadership and future leaders in BSSR starting at the undergraduate level, a distinct approach SOAR has taken.

### Program limitations

No program is without limitations. SOAR was located in an elite university with a highly ranked school of public health, which may limit its transferability or generalizability in other university or college settings. While the high success of SOAR scholars matriculating into graduate school and number of peer reviewed publications and presentations suggests increased social science identity and self-efficacy, we do not have evidence of longstanding effects. Although not reflective of students in the program, SOAR students attend an elite public research university where over 50% of the student body comes from households of the top 20% of income earners. Some students arrived in SOAR having experienced feelings of isolation, questions of worthiness, and uncertainty about their potential to pursue a post-graduate degree. The SOAR program actively works to challenge and refute conceptions of inferiority and competition. Yet, potential mitigation of feelings of inferiority and competition among SOAR students may be difficult to maintain as SOAR scholars matriculate into similarly elite graduate schools. This challenge may be even more relevant to SOAR scholars considering they reflect historically underrepresented groups in HIV- related BSSR.

### Conclusion

SOAR is a program focused on creating a pipeline of HIV-related BSSR scholars starting at the undergraduate level, many of whom will be a part of the future public health workforce and lead the field to address new public health challenges. While the focus of SOAR is on HIV-related BSSR, the programmatic approach can be applied to other public health issues. In a period where public health is both increasingly in the limelight and being defunded, including within the SOAR program, it is imperative that public health and future leadership development capitalize on the increased attention to teach public health and identify future public health leaders.

SOAR’s success is rooted in acknowledging and embracing difference, identifying key opportunities to promote cohesion, and demonstrating deep care for individuals and the collective cohort. The concrete research skills that are developed through SOAR may lay the foundation for a successful model to develop future public health leaders. Key lessons highlighted above demonstrate an ability to not only engage students while in SOAR but also grow the SOAR program over time which may suggest that an interdisciplinary, HIV-focused, early public health leadership program is a successful model to develop future leaders. By dedicating resources either through nonprofit and philanthropic funding or galvanizing experienced public health leaders in diverse sectors, investing in undergraduates *in addition* to graduate students will build an increasingly critical, sustainable, and diverse base of public health leaders to address the complex, and ever-changing public health landscape in the United States.

## Data Availability

The original contributions presented in the study are included in the article/supplementary material, further inquiries can be directed to the corresponding author.
